# Methylatable Signaling Helix Coordinated Inhibitory Receiver Domain in Sensor Kinase Modulates Environmental Stress Response in *Bacillus Cereus*

**DOI:** 10.1371/journal.pone.0137952

**Published:** 2015-09-17

**Authors:** Jung-Chi Chen, Jyung-Hurng Liu, Duen-Wei Hsu, Jwu-Ching Shu, Chien-Yen Chen, Chien-Cheng Chen

**Affiliations:** 1 Department of Biotechnology, National Kaohsiung Normal University, Kaohsiung, Taiwan; 2 Institute of Genomics and Bioinformatics, National Chung Hsing University, Taichung, Taiwan; 3 Agricultural Biotechnology Center (ABC), National Chung Hsing University, Taichung, Taiwan; 4 Department of Medical Biotechnology and Laboratory Science, Chang Gung University, Tao-Yuan, Taiwan; 5 Department of Earth and Environmental Sciences, National Chung Cheng University, Chiayi, Taiwan; Chang-Gung University, TAIWAN

## Abstract

σ^B^, an alternative transcription factor, controls the response of the cell to a variety of environmental stresses in *Bacillus cereus*. Previously, we reported that RsbM negatively regulates σ^B^ through the methylation of RsbK, a hybrid sensor kinase, on a signaling helix (S-helix). However, RsbK comprises a C-terminal receiver (REC) domain whose function remains unclear. In this study, deletion of the C-terminal REC domain of RsbK resulted in high constitutive σ^B^ expression independent of environmental stimuli. Thus, the REC domain may serve as an inhibitory element. Mutagenic substitution was employed to modify the putative phospho-acceptor residue D827 in the REC domain of RsbK. The expression of RsbK_D827N_ and RsbK_D827E_ exhibited high constitutive σ^B^, indicating that D827, if phosphorylatable, possibly participates in σ^B^ regulation. Bacterial two-hybrid analyses demonstrated that RsbK forms a homodimer and the REC domain interacts mainly with the histidine kinase (HK) domain and partly with the S-helix. In particular, co-expression of RsbM strengthens the interaction between the REC domain and the S-helix. Consistently, our structural model predicts a significant interaction between the HK and REC domains of the RsbK intradimer. Here, we demonstrated that coordinated the methylatable S-helix and the REC domain of RsbK is functionally required to modulate σ^B^-mediated stress response in *B*. *cereus* and maybe ubiquitous in microorganisms encoded RsbK-type sensor kinases.

## Introduction

Microorganisms experiencing environmental fluctuations commonly exhibit a short-lived, reversible response through the tight coordination of dedicated sets of sensory modules to increase cell survival and recovery [1]. Some alternative sigma factors encoded in most bacteria that target recognizable sequences control specialized regulons under specific conditions [2]. For example, the well-studied stress responsive alternative sigma factor σ^B^ found in low-GC Gram-positive bacteria such as *Bacillus subtilis*, *Staphylococcus aureus*, and *Listeria monocytogenes* controls σ^B^-dependent regulon expression mediated by signaling cascades to cope with a variety of environmental stresses, including changes in temperature, pH, ethanol levels and osmolarity [3]. σ^B^ not only participates in physiologically relevant responses to multiple environmental stresses but also plays an important role in virulence in the bacteria *Bacillus anthracis* [4], *Listeria monocytogenes*, and *Staphylococcus aureus* [5, 6] as well as in other high G-C Gram-positive bacteria, including *Mycobacterium tuberculosis*, albeit to a lesser extent, and *Streptomyces* species [7]. In *Streptomyces coelicolor*, σ^B^ works in concert with several paralogous sigma factors, constituting a complex network with functions in osmotic and oxidative stress responses, cellular differentiation, and antibiotics production [8–10].

The activation mechanism paradigm of σ^B^ is established in the low-GC model *B*. *subtilis* through the interplay of eight regulatory proteins, namely *rsbR*, *rsbS*, *rsbT*, *rsbU*, *rsbV*, *rsbW*, *sigB*, and *rsbX*, encoded in the *sigB* operon [11, 12]. The basic core theme of the partner-switching mechanism refers to the protein-protein interactions that lead to the formation of either stable RsbW/σ^B^ or RsbV/RsbW complexes; these interactions are critically determined by the alternating phosphorylation and dephosphorylation of RsbV anti-anti-σ factor [13–15]. In unstressed cells, RsbV is phosphorylated on a conserved serine residue, and the phosphorylation renders RsbV inactive. RsbW, an anti-σ factor, is then free to sequester σ^B^ and prevent its association with RNA polymerase. Two homologous PP2C phosphatases, RsbU and RsbP, are activated in response to physical stress or energy stress, respectively, to dephosphorylate RsbV and force it to form a complex with RsbW, thereby releasing σ^B^ [16–18]. The association of RsbR and its homologues with RsbS and dissociable RsbT forms a supramolecular stressosome that functions as the signaling hub and integrates multiple physical stress signals for the activation of σ^B^ [19]. RsbT released from stressosome upon stress stimulates RsbU to dephosphorylate phosphorylated RsbV.

In the human pathogen *B*. *cereus*, which is closely related to *B*. *subtilis*, σ^B^ activation is accomplished by coupling the basic core theme of the partner-switching mechanism comprising RsbV/RsbW/σ^B^ to a two-component system (TCS) sensory module, which comprises the transmembrane sensor histidine kinase of σ^B^-mediated stress response RsbK, the cognate response regulator (RR) RsbY and the methyltransferase RsbM, which can specifically methylate and thereby negatively regulate RsbK [20]. The RsbK-M-Y mode is important for the regulation of a broad range of functions and is found widespread in microorganisms [20]. Although RsbY exhibits similar PP2C phosphatase activity to that of RsbU, the amino acid sequence of the N-terminal REC domain of RsbY that is homologous to CheY is considerably variable compared with that of RsbU, which is mainly associated with the regulator RsbT. This variability indicates distinct upstream signaling routes among *B*. *subtilis* and *B*. *cereus*. In the TCS scenario, upon perceiving environmental stimuli, the HK domain of the sensor kinase catalyzes the autophosphorylation of a conserved histidine residue in the H-box, and the phosphate on phosphorylated histidine is transferred to the downstream RR either by direct phosphoryl transfer or by histidine-containing phosphotransferase (Hpt) protein-mediated multi-step phosphoryl transfer (His-Asp-His-Asp) [21–23]. In most cases, phosphorylation of the REC domain of the response regulator (RR) in turn modulates the binding to specific sites on DNA to alter gene expression or other regulatory functions of the coupled output domain involved in adaptive responses [24, 25].

The predominant RsbK-M-Y regulatory module largely focuses on protein-protein interactions among regulator proteins that modulate σ^B^ activity. RsbK is required to trigger the σ^B^ activation pathway [26], and the structural architecture of multiple functional domains reveals that RsbK is a typical TCS hybrid sensor kinase [27]. However, our understanding of the roles of RsbK subdomains upon stimulation remains limited. Particularly, the function of the REC domain and why methylation of the signaling helix is required to inhibit σ^B^ prior to stress require further investigation. In this study, using genetic approaches and structural simulation, the C-terminal REC domain of RsbK was shown to play an important role on σ^B^ activity. Furthermore, the subtle interaction between the REC subdomain and the methylated signaling helix appeared to be necessary with respect to σ^B^ activation.

## Materials and Methods

### Bacterial strains and plasmids

The genotypes and sources of the bacterial strains and plasmids used in this study are listed in S1 Table. *Bacillus cereus* (*B*. *cereus*; ATCC 14579) was grown in brain heart infusion (BHI) medium at 30°C, and *Escherichia coli* (*E*. *coli*) was cultured in Luria-Bertani (LB) medium at 37°C. The *E*. *coli* strains DH5-α, XL-1 blue and BL21 (λDE3) were used for plasmid manipulation and IPTG-induced expression. Growth of the cultures was monitored by measuring the optical density at 600 nm (OD_600_). The antibiotic selection used for cloning and mutant screening was performed with ampicillin (50 μg/ml), erythromycin (3 μg/ml), and spectinomycin (100 μg/ml), as required.

### Bacterial two-hybrid assay

Bacterial two-hybrid (BACTH) analysis of protein or subdomain interactions was performed with a bacterial two-hybrid system as previously described [28]. To construct recombinant plasmids to analyze interactions between the REC domain and other subdomains, *rsbK*, subdomain-truncated *rsbKs* and the coding region of the REC domain of *rsbK* were amplified by PCR using specific oligonucleotide primers (S2 Table). The REC domain coding region amplicon was cloned into pKT25, and the other amplicons were individually cloned in-frame into the multiple cloning sites (MCSs) of pUT18. To analyze the interaction of RsbK with itself, full-length *rsbK* was also cloned into pKNT25. The complementation of recombinant plasmid pairs was indicated by blue colony growth on M63-defined medium/maltose containing 0.5 mM isopropyl-β-D-thiogalactopyranoside (IPTG) and 40 μg/ml 5-bromo-4-chloro-3-indolyl-β-D-galactopyranoside (X-gal). Both media contained ampicillin and kanamycin to select for the inserted plasmids. The plates were incubated at 30°C for a maximum of 40 h. The pKT25-*zip* and pUT18-*zip* plasmids were used as positive controls for protein interactions, and the empty vectors pKT25, pKNT25 and pUT18 were used as negative controls.

To measure β-galactosidase activity in a bacterial assay, exponentially growing cells (2 ml) were harvested and resuspended in 0.5 ml of Z buffer [0.06 M Na_2_HPO_4_, 0.04 M NaH_2_PO_4_, 0.01 M KCl, 1 mM MgSO_4_, 1 mM dithiothreitol (DTT)]. The cells were disrupted with glass beads (212 to 300 μm; Sigma) in a Fast Prep 120 homogenizer (Savant), and the cell extract was obtained after centrifugation. Next, 0.7 ml of Z buffer and 200 μl of 2 mg ml^−1^ 2-nitrophenyl-β-D-galactoside (Sigma) for the β-galactosidase assay were added to 100 μl of cell extract. The mixture was incubated at 37°C, and the reaction was stopped by the addition of 0.2 ml of 2 mM Na_2_CO_3_. Subsequently, the optical density of the reaction mixture was measured at 405 nm. The protein content was measured using the Bio-Rad protein assay with bovine serum albumin as the standard. Specific activities are expressed in the units of β-galactosidase per milligram of protein (Miller units) using the following formula: [O.D.405 nm x 378]/[time (min) x volume of cell extract (ml) x protein concentration (mg/ml)].

### Construction of the *rsbKM*-null strain and complementary plasmids

**Fig 1:**
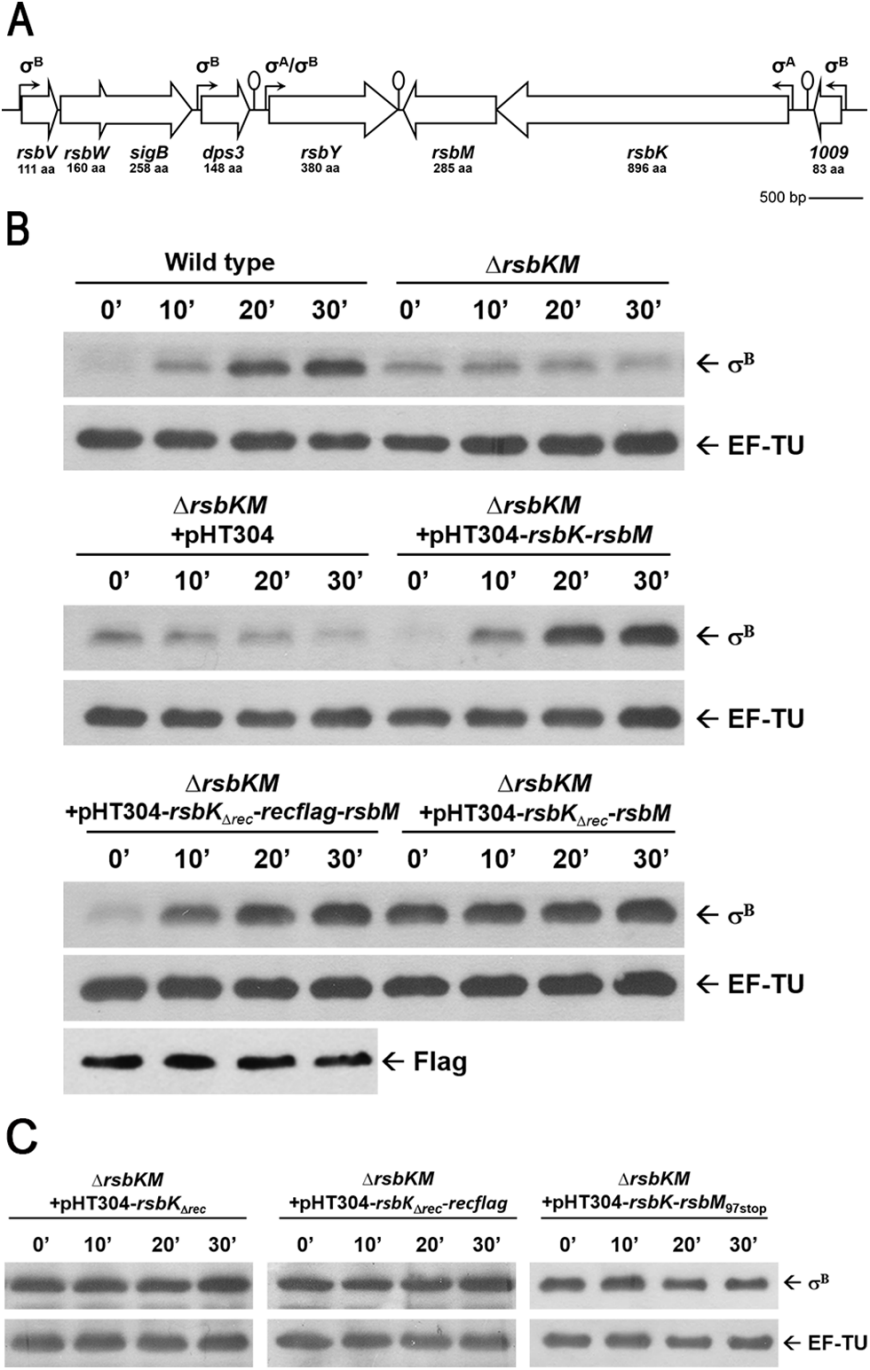
Functional analyses of the REC domain of RsbK. (A) Schematic diagram of the genetic organization of the *sigB* cluster. The *sigB* cluster is composed of seven genes in which *rsbK* and *rsbM* constitute an operon. (B) The REC domain truncation of RsbK resulting in constitutive high σ^B^. *B*. *cereus* Δ*rsbKM* complemented with indicated plasmids were grown to the mid-exponential growth phase at 30°C and then exposed to mild heat shock (42°C). Immunoblotting was used to analyze σ^B^ levels and confirm the protein stability of Flag-REC. (C) Coordination of the REC domain of RsbK and RsbK methylation. *B*. *cereus* Δ*rsbKM* was complemented with pHT304-*rsbK*_Δrec_, pHT304-*rsbK*_Δrec_-*recflag* and pHT304-*rsbK*-*rsbM*_97stop_. σ^B^ expression level was analyzed with Western blotting as described. EF-TU was used as an input.

Gene organization of the *sigB* cluster is shown in Fig 1A. *rsbK-rsbM* gene deletion was performed as previously described [29]. To produce the pMAD-Δ*rsbKM* plasmid, a 739 bp DNA fragment beginning 424 bp upstream of *rsbK* and continuing through 315 bp of the 5’ coding region, and an additional 999 bp DNA fragment directly downstream of *rsbM* were amplified from *B*. *cereus* genomic DNA and cloned into the pMAD vector. The coding region of the spectinomycin resistance gene (1221 bp) from pDG1728 was then inserted into either end of two inserted DNA fragments listed above. The oligonucleotide primers used are shown in S2 Table, and the plasmid map is indicated in S1A Fig.

The pMAD-Δ*rsbKM* plasmid was introduced into *B*. *cereus* by electroporation at 1.7 kV in 1-cm electrocuvettes (Eppendorf). Transformants were obtained after culturing for 2 days at 30°C on BHI plates containing spectinomycin, erythromycin, and X-gal. A pool of individual transformants was used to inoculate a culture of BHI medium without antibiotics, and this culture was then incubated at 40°C until the stationary growth phase was reached. Two additional growth cycles were performed by diluting the stationary phase culture into fresh media, and single colonies were isolated by plating dilutions of the culture onto BHI plates containing X-gal and spectinomycin overnight. Several white colonies were also isolated to verify the erythromycin-sensitive phenotype. DNA was purified from several clones and analyzed by PCR using oligonucleotides that hybridized outside of the inserts to verify the presence of the deletion and the insertion of the spectinomycin resistance gene. Gene deletion was also confirmed by Southern blotting.

To construct the complementary plasmid pHT304-*rsbK-rsbM* (S1B Fig), a DNA fragment (2255 bp), containing a partial sequence from *bc1009* and internal to *rsbK* with an endogenous BamHI site, and another DNA fragment (1782 bp), which included the remaining *rsbK* sequence and full-length *rsbM*, were amplified by PCR from the genomic DNA of *B*. *cereus* using the primers listed in S2 Table. Both DNA fragments were restricted and then inserted into pHT304 to generate pHT304*-rsbK-rsbM*. In addition, a DNA fragment obtained from the above-described 1782 bp DNA fragment lacking the REC domain coding region corresponding to the 759^th^-896^th^ amino acid sequence was used instead of original the 1782 bp DNA fragment to generate the complementary plasmid pHT304-*rsbK*_Δrec_ (S1B Fig). Moreover, a DNA fragment that included the Flag-tagged REC domain downstream of an additional 25 nucleotides from the DNA sequence located between *rsbK* and *rsbM*, which carries a translation initiation site and allows for trans expression, was inserted followed by *rsbK*_Δrec_ to construct pHT304-*rsbK*_Δrec_-*recflag* (S1B Fig). Two other complementary plasmids, pHT304-*rsbK*_Δrec_-*rsbM* (S1B Fig) and pHT304-*rsbK*_Δrec_-*recflag*-*rsbM* (S1B Fig), were derived from the parental plasmids pHT304-*rsbK*_Δrec_ and pHT304-*rsbK*_Δrec_-*recflag* by inserting *rsbM*. All restriction enzymes used in the construction of complementary plasmids were denoted.

The complementary plasmid pHT304-*rsbK-rsbM*_97stop_ (S1B Fig) was derived from pHT304-*rsbK-rsbM*, except the codon (AAA) of the internal 97^th^ lysine residue (K97) in the *rsbM* coding region was replaced by a stop codon (TAA). The pHT304-*rsbK*_D827N_-*rsbM* and pHT304-*rsbK*_D827E_-*rsbM* plasmids were derived from pHT304-*rsbK-rsbM* using site-directed mutagenesis, performed as previously described [30, 31] with slight modifications, to generate the vector carrying the D827-mutant *rsbK*. A standard PCR reaction (50 μl) was conducted in Phusion^TM^ GC buffer containing pHT304*-rsbK-rsbM* (30 ng) as the template and 1 U Phusion^TM^ high-fidelity DNA polymerase (NEB) in 3% DMSO using the primer pairs listed in S2 Table. The PCR product was digested with the restriction enzyme DpnI to cleave the methylated pHT304*-rsbK-rsbM* template and was then transformed into the *E*. *coli* XL-1 blue strain. The mutagenized plasmid was extracted, and the mutations were confirmed by DNA sequencing.

### The construction of pET21b-*rsbK*, pET21b-*rsbK*_D827N_, pET21b-*rsbK*_D827E_, and pET21b-*rsbK*-*rsbM*-*6xHis*

The entire *rsbK* coding region was PCR amplified from the genomic DNA of *B*. *cereus* using the primers pET21b-*rsbK*-NdeI-F and pET21b-*rsbK*-XhoI-R. The PCR product was digested by NdeI and XhoI and cloned into the plasmid pET21b to generate the pET21b-*rsbK* plasmid (S1 Table). Site-directed mutagenesis with Phusion^TM^ high-fidelity DNA polymerase was employed to construct the pET21b-*rsbK*_D827N_ and pET21b-*rsbK*_D827E_ plasmids using pET21b-*rsbK* as the template. In addition, the full-length reading frame of the *rsbK*-*rsbM* operon with the deleted stop codon was cloned into the expression vector pET21b to construct pET21b-*rsbK*-*rsbM*-*6xHis* (S1 Table). For this purpose, the DNA fragment from the first codon of *rsbK* to the region containing the only intrinsic BamHI restriction site of *rsbK* and another DNA fragment of the sequence from the intrinsic BamHI restriction site to the terminator of *rsbM* were amplified using primer pairs with restriction sites introduced (S2 Table). Subsequently, the two PCR products were digested with NdeI/BamHI and BamHI/XhoI, respectively, and were inserted into pET21b.

### Western blotting

*B*. *cereus* and its engineered derivatives were grown in BHI broth at 30°C with aeration until an OD_600_ of 0.5 was reached. They were then incubated at 42°C for 10, 20, and 30 min. The cells were pelleted by centrifugation and immediately frozen in liquid nitrogen. The cells were subsequently disrupted by sonication. Cell extracts (30 μg) were separated by SDS-PAGE and transferred onto nitrocellulose membranes. The membranes were probed with 1:5,000 dilutions of anti-σ^B^ and anti-EF-TU (BC0129) polyclonal antisera in PBS-Tween at room temperature for 1 h with gentle agitation. The membranes were then incubated with a secondary antibody (HRP-conjugated goat anti-rabbit IgG). For the detection of His-tagged RsbK expression in *E*. *coli*, the membranes were blotted with a 1:2,000 dilution of an anti-His tag monoclonal antibody followed by incubation with a secondary antibody (HRP-conjugated goat anti-mouse IgG). Enhanced chemiluminescence (ECL) detection reagents were added to develop the images.

### Protein overexpression and purification

For the purification of RsbM, *E*. *coli* BL21 (λDE3) cells were transformed with pET21b-*rsbK*-*rsbM*-*6xHis*. Transformed cells were inoculated in 250 mL of LB medium containing 50 mg/ml ampicillin and grown at 37°C with vigorous shaking. At an OD_600_ of 0.6, protein expression was induced for 3 h by adding IPTG to a final concentration of 0.5 mM. Subsequently, the culture was chilled on ice, and the cells were harvested. The recombinant His_6_-fused RsbM protein was then purified. Briefly, harvested cells were suspended in 30 ml of cell lysis buffer [50 mM Tris–HCl (pH 8.5), 10 mM EDTA, 5 mM DTT, and 1 mM phenylmethanesulfonylfluoride] and sonicated. Soluble proteins were separated from the cell debris by centrifugation at 15000 x *g* for 30 min at 4°C, and the supernatant was passed through a 0.22-μm filter (Millipore) and loaded onto a nickel column (GE Healthcare). Bound proteins were eluted according to the manufacturer’s instruction.

### *In vitro* methylation assay

For the overproduction of RsbK, *E*. *coli* BL21 (λDE3) cells were transformed with pET21b-*rsbK*. As previously described, the cells were harvested by centrifugation at 6000 x *g* for 10 min at 4°C after IPTG induction, washed with cold PBS and then re-suspended in methylation buffer (50 mM HEPES [pH 8.0], 0.01% [v/v] NP-40, 10 mM NaCl, 1 mM DTT, 1 mM PMSF). The collected cells were disrupted by sonication to prepare whole cell lysates, and 100 μg of each cell lysate was incubated with purified recombinant RsbM protein in 45 μl methylation buffer supplemented with 500 nCi of S-adenosyl-L-(methyl-^14^C) methionine (^14^C-SAM; 61 mCi/mmol, GE Healthcare) (radioactive methylation) for 1 h at room temperature. The reactions were stopped by adding 3X 10% SDS-PAGE sample buffer (187.5 mM Tris-HCl, 6% (w/v) SDS, 10% glycerol, 0.03% (w/v) bromophenol blue, 1.25 M DTT, pH 6.8) followed by heating at 95°C for 10 min. The samples were analyzed by 12% SDS-PAGE and stained with Coomassie blue. The gel was treated with Amplify Fluorographic Reagent (GE Healthcare) for 30 min, according to the manufacturer’s instruction, and dried by a vacuum. The radioactivity was visualized by exposing the gel to an X-ray film at -80°C for one week.

### Sequence alignment and molecular modeling

Amino acid sequences of the RsbK homologs were extracted from NCBI GenBank using PSI-Blast [32]. These sequences were first processed with CH-HIT [33] to remove redundant sequences before being used in the alignment. Sequences with 90% or higher identities were grouped in one cluster, and only one representative from each group was selected for the alignment. Information about these representative sequences is summarized in S3 Table. The multiple sequence alignment was performed using the default parameters of MUSCLE [34] and was then rendered by Jalview [35].

Structures of the RsbK cytoplasmic domains HK and REC were created using MODELLER 9v12 [36]. The crystal structure of the complex formed by *Thermotoga maritima* class I HK853 and its cognate response regulator, RR468 (PDB ID: 3DGE) [37], was used as the template structure. The target–template alignment was generated by hidden Markov model matching using the Phyre2 server [38]. To sample the dynamics at the protein-protein interface resulting from side-chain conformational changes, from 100 models generated from the modeling process, we selected five models with the best discrete optimized protein energy (DOPE) scores for protein-protein interaction analysis. The models were further optimized by energy minimization using the Smart Minimizer method with the Generalized Born implicit solvation model in Discovery Studio 2.5 (Accelrys, USA). CHARMm force field was used in the simulation. The model quality was assessed using PROCHECK [39] and the QMEAN Z-score [40] in the SWISS-MODEL Workspace [41]. The strengths of protein-protein interactions were evaluated using PISA [42]. The protein model images in the figures were drawn using PyMOL (Schrödinger, LLC, USA).

## Results

### The REC domain of RsbK may serve as an inhibitory element

The gene organization shows that RsbK and RsbM constitute an operon in the *sigB* cluster in *B*. *cereus* (Fig 1A) in which RsbM acts as a negative regulator to methylate the hybrid sensor RsbK [20]. RsbK comprises several domains, including CHASE3, HAMP, GAF, S-helix, HK, CA domains and a C-terminal REC domain whose function remains not clear. To explore the role of the REC domain of RsbK, we generated a *B*. *cereus rsbKM* deletion mutant (S1A Fig) and constructed various complementary plasmids, pHT304-*rsbK-rsbM*, pHT304-*rsbK*_Δrec_ *-rsbM* and pHT304-*rsbK*_Δrec_ *-recflag-rsbM* (S1B Fig). These low copy plasmids were thought to express three protein combinations, including RsbK and RsbM, the REC-deleted RsbK and RsbM, and the REC-deleted RsbK and RsbM and the Flag-tagged REC protein expressed in *trans*, respectively. As expected, the *rsbKM* deletion mutant was defective in its response to 42°C heat stress; however, the *rsbKM* mutant complemented with pHT304-*rsbK-rsbM* could induce σ^B^ upon 42°C heat stress at a level comparable to the wild-type strain (Fig 1B). Intriguingly, the introduction of pHT304-*rsbK*_Δrec_ *-rsbM* into the *rsbKM* deletion mutant resulted in constitutive high σ^B^, whereas the introduction of pHT304-*rsbK*_Δrec_ *-recflag-rsbM* restored the phenotype of heat stress-inducible σ^B^ (Fig 1B). This result demonstrated that loss of the REC domain led to σ^B^ activation independently of environmental stress, and the phenotype of constitutive high σ^B^ can be rescued by complementation with the in *trans* expressed Flag-REC protein. Our data strongly suggest that the REC domain may serve as an inhibitory element to prevent σ^B^ activation prior to environmental stress.

In addition to the inhibitory function of the REC domain, methylation of the S-helix of RsbK by RsbM has been shown essential to repress σ^B^ [20]. It needs to investigate whether the REC domain and methylation of the S-helix repress σ^B^ independently or function in a sequential manner. To address this, three complementary plasmids including pHT304-*rsbK*_Δrec_, pHT304-*rsbK*_Δrec_ *-recflag* and pHT304-*rsbK*-*rsbM*_97stop_ were constructed and then introduced into the *rsbKM* deletion mutant (S1B Fig). The former two plasmids lack the *rsbM* gene compared with pHT304-*rsbK*_Δrec_ *-rsbM* and pHT304-*rsbK*_Δrec_ *-recflag-rsbM* (S1B Fig), and the latter is expected to express a malfunctioning truncated RsbM mutant. The introduction of these three plasmids into the *rsbKM* deletion mutant resulted in constitutive high σ^B^ (Fig 1C), indicating that the REC domain per se was insufficient to repress σ^B^ in the absence of RsbM. In other words, the REC domain appears to work in concert with RsbK methylation. We proposed that RsbM-mediated methylation at specific residue(s) on the S-helix is required to recruit the REC domain and thereby repress σ^B^ before environmental stress.

### Subdomain interactions in RsbK analyzed by BACTH

**Fig 2:**
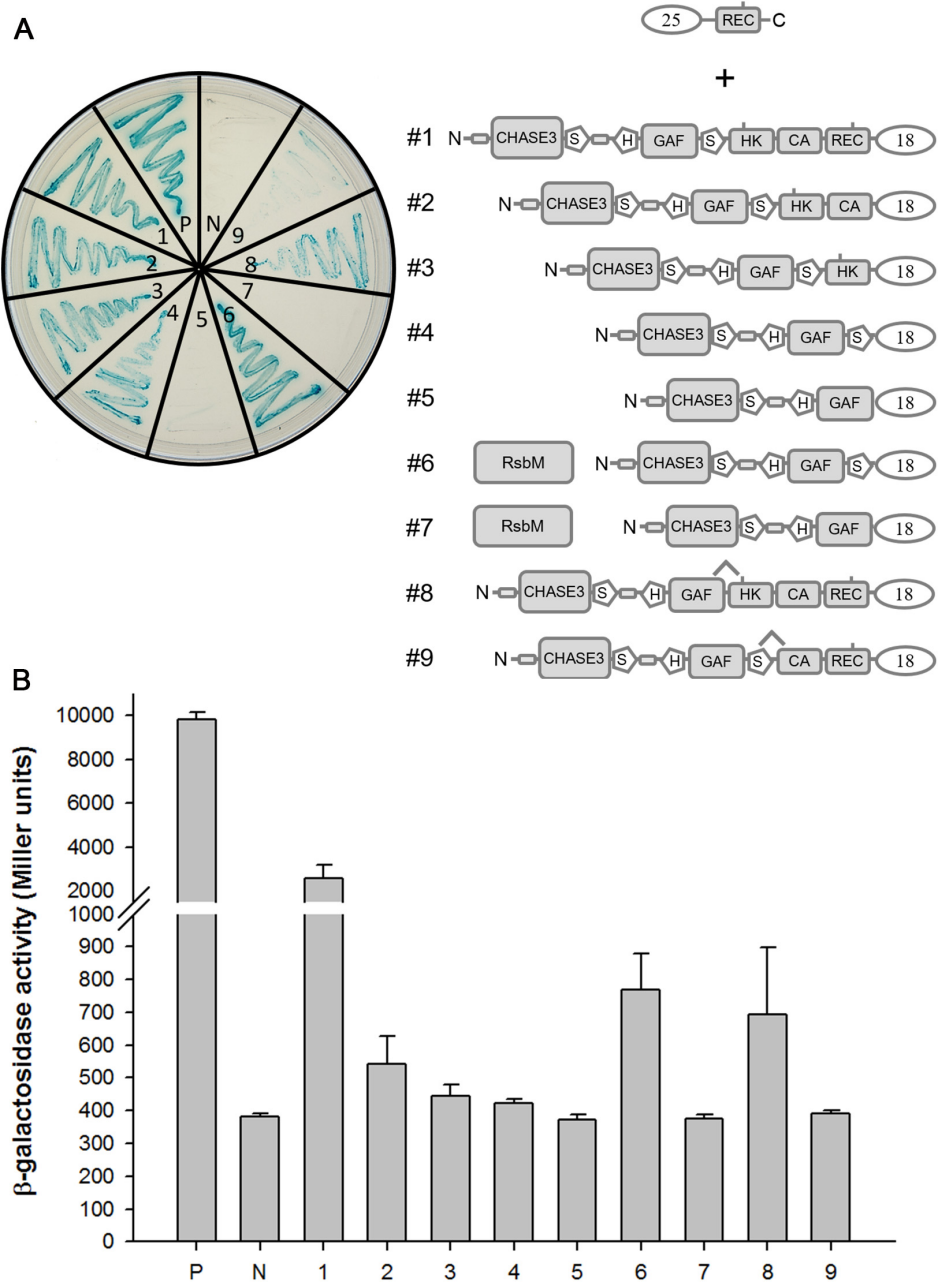
Analyses of subdomain interactions using the bacterial two-hybrid system. The diagram shows various pairs of pKT25-*rec* and pUT18-derived constructed plasmids. *E*. *coli* BTH101 cells containing various combinations of plasmid constructs depicted as different numbers were propagated on an M63/maltose X-Gal indicator plate (A). Subdomain interaction strength was indicated by β-galactosidase activity with statistical analysis (B). Error bars indicate the standard deviation derived from six independently grown cultures. “P” denotes the pUT18-*zip* and pKT25-*zip* as positive controls; “N” represents the pUC18 and pKT25 vectors, used as negative controls.

To test the above hypothesized mechanism, BACTH was employed to analyze whether the REC domain interacted with other RsbK subdomains, particularly the S-helix. The pKT25 plasmid containing the REC domain was constructed, and full-length RsbK or RsbK sequences with successive subdomain truncations such as REC-truncated RsbK, REC-CA-truncated RsbK, REC-CA-HK-truncated RsbK, REC-CA-HK-S-truncated RsbK and RsbK with only HK or S-helix deletion were separately constructed in pKT18 plasmids (Fig 2A). The physical interaction strength between the REC domain and the respective RsbK truncated proteins was indicated by *E*. *coli* growth of blue colonies on M63 selection medium and quantification of β-galactosidase activity. Bacterial growth were comparable to measurements of β-galactosidase activity showing that the REC domain could interact with full-length RsbK (Fig 2-#1), REC-truncated RsbK (Fig 2-#2), REC-CA-truncated RsbK (Fig 2-#3), REC-CA-HK-truncated RsbK (Fig 2-#4) and RsbK with S-helix deletion (Fig 2-#8). However, the interaction strength decreased in the case of the REC domain and RsbK with HK deletion (Fig 2-#9). Moreover, the REC domain failed to interact with REC-CA-HK-S-truncated RsbK (Fig 2-#5). These data suggest that the REC domain interacts mainly with the HK domain and partly with the S-helix within full length RsbK. Interestingly, when RsbM was co-expressed with the REC domain and REC-CA-HK-truncated RsbK, the interaction strength between the REC domain and REC-CA-HK-truncated RsbK was higher compared to no RsbM co-expression (Fig 2-#4, #6). On contrast, no interaction was observed between the REC domain and REC-CA-HK-S truncated RsbK despite RsbM was co-expressed (Fig 2-#5, #7). This result suggests that RsbM appeared to strengthen the interaction between the REC domain and S-helix. Notably, lower β-galactosidase activity implicated weak subdomain interactions, if interactable, probably due to prominent conformational change in the test conditions. We further examined whether the REC domain interacts with the sole HK or S-HK domain. Neither the sole HK domain nor the S-HK domain can interact with the REC domain. This result indicates that the N-terminal extension from CHASE3 to the GAF domain is also crucial to maintain the proper conformation for the REC domain to interact with the S-HK domain (data not shown). Western blotting using an anti-His tag monoclonal antibody confirmed the similar expression of his-tagged RsbM in *E*. *coli* BHT101 cells (data not shown).

### D827 in the REC domain involved in σ^B^ regulation

**Fig 3:**
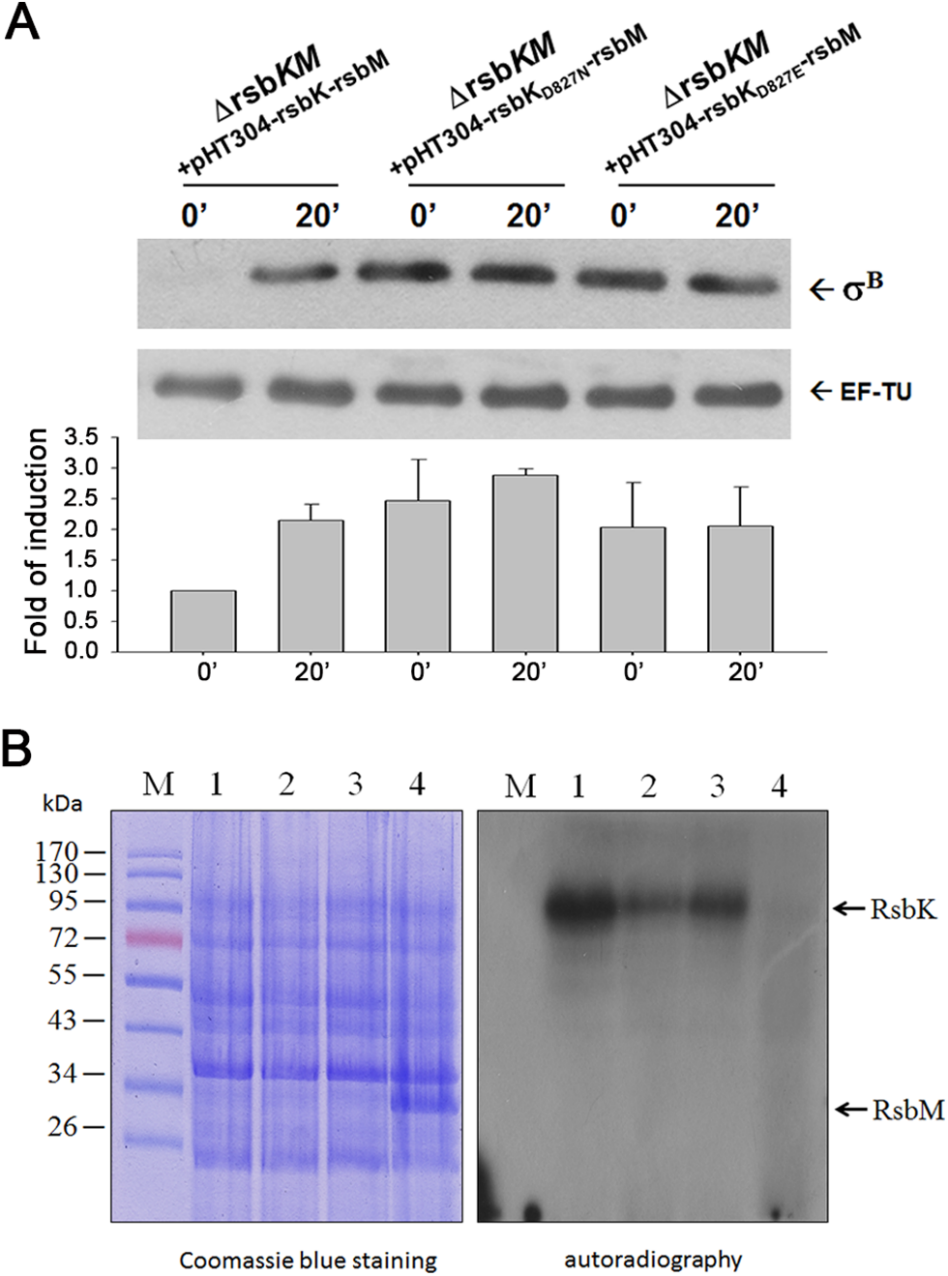
Effect of RsbK D827 variants on σ^B^. (A) RsbK D827 variants resulting in constitutive high σ^B^. The *rsbKM* deletion mutants loaded with RsbK, RsbK_D827N_ and RsbK_D827E_ were exposed to 42°C heat shock and harvested at various time intervals. Western blot from SDS–PAGE analysis was used to quantify the σ^B^ expression levels, normalized to the level of EF-TU via ImageJ 1.74v software. In all cases the starting signal prior to the stress is denoted as 1. Values are mean ± standard deviation from three independent experiments. (B) The methylation of RsbK D827 variant proteins by purified RsbM *in vitro*. The *E*. *coli* cell lysates loaded with overexpressed His-tagged RsbK variant proteins were incubated with the purified RsbM in the presence of ^14^C-SAM. The mixtures were resolved by 12% SDS-PAGE followed by Coomassie blue staining (left), and the gel was then subjected to autoradiography (right). Purified RsbM was separately incubated with cell lysate containing RsbK (lane 1), RsbK_D827N_ (lane 2), RsbK_D827E_ (lane 3), co-expressed RsbK and RsbM (lane 4). RsbK D827 variant proteins and RsbM are indicated by arrows. M is denoted as marker proteins.

Sequence analysis showed that RsbK contains a conserved histidine residue (H505) in the HK domain and an aspartate residue (D827) predicted as a putative phospho-acceptor in the C-terminal REC domain. We examined whether D827 regulates σ^B^ in response to environmental stress. Two complementary plasmids, pHT304-*rsbK*_D827N_-*rsbM* and pHT304-*rsbK*_D827E_-*rsbM*, carrying RsbK mutants with site-directed mutagenesis at D827 were generated (S1 Table). Residue substitutions of RsbK_D827N_ and RsbK_D827E_ were used to mimic non-phosphorylated and phosphorylated D827, respectively [43, 44]. The *rsbKM* deletion strain was introduced along with the complementary plasmids pHT304-*rsbK-rsbM*, pHT304-*rsbK*_D827N_-*rsbM* and pHT304-*rsbK*_D827E_-*rsbM*, and these resulting strains were subsequently exposed to 42°C followed by Western blot to analyze σ^B^ levels. Consequently, compared with the inducible σ^B^ upon 42°C heat stress in the strain harboring pHT304-*rsbK-rsbM*, the introduction of pHT304-*rsbK*_D827N_-*rsbM* and pHT304-*rsbK*_D827E_-*rsbM* in the *rsbKM* deletion strain led to constitutive σ^B^ activation (Fig 3A). Our result indicates that the state of D827, if phosphorylatable, might be involved in σ^B^ activation upon environmental stress.

Although either RsbK_D827N_ or RsbK_D827E_ constitutively activates σ^B^ (Fig 3A), it is possible that these two RsbK D827 variants activate σ^B^ via preventing RsbK from being methylated. To test this possibility, the recombinant protein wild-type RsbK, RsbK_D827N_ or RsbK_D827E_ in *E*. *coli* cell lysates was incubated with purified RsbM in the presence of C^14^-labelled SAM *in vitro*. The wild-type RsbK and the two RsbK D827 variants can be methylated (Fig 3B, lane 1–3). Whereas, RsbK purified from *E*. *coli* co-expression with RsbM was no more methylated by the purified RsbM with C^14^-labelled SAM *in vitro* because RsbK had been fully methylated by co-expressed RsbM in *E*. *coli* BL21 (Fig 3B, lane 4). This result indicates that change of the state of D827 did not abolish RsbM-specific methylation of RsbK.

### Formation of RsbK homodimers

**Fig 4:**
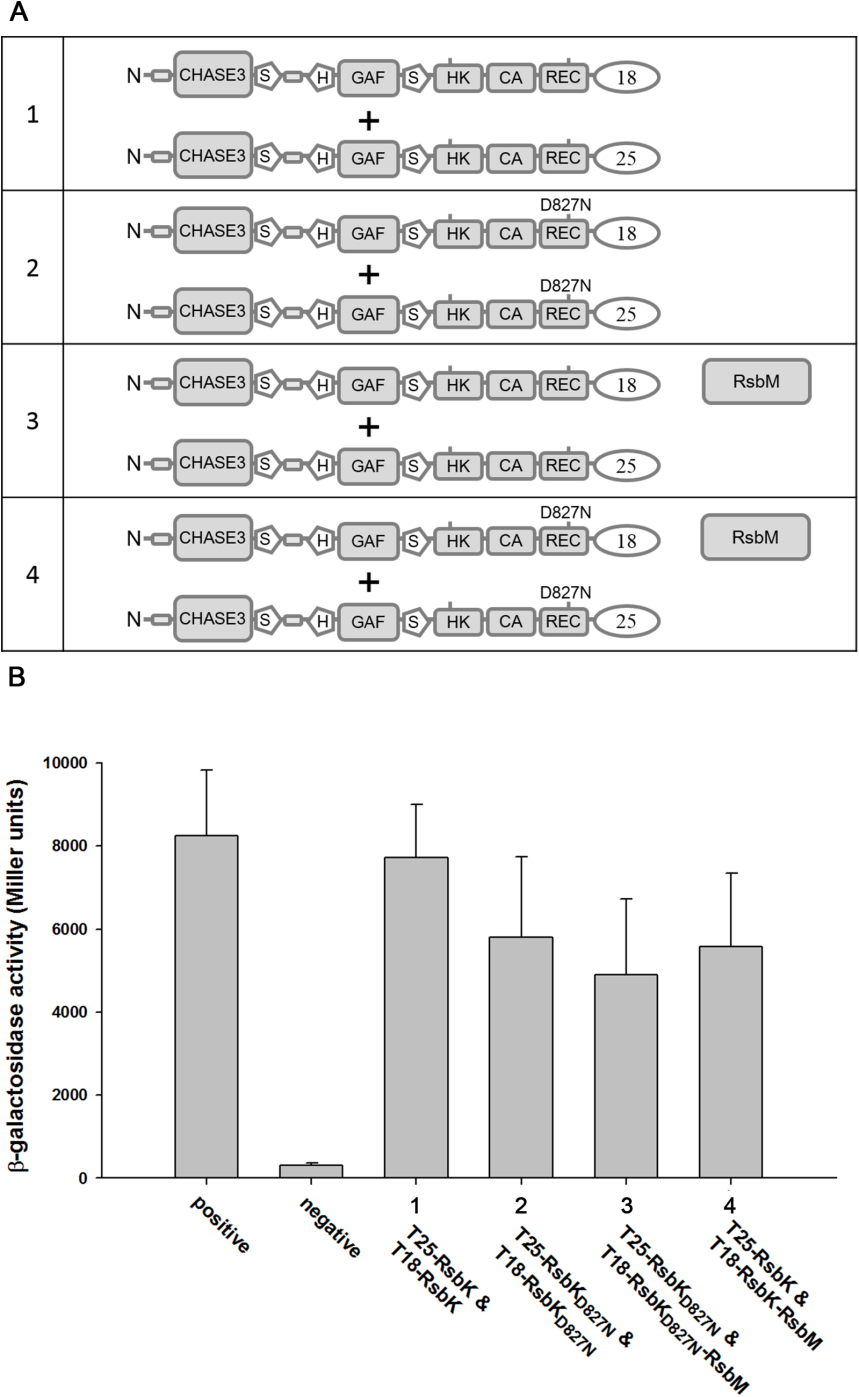
Formation of the RsbK homodimer. The *rsbK* gene was cloned into the reporter plasmids pKT18 and pKNT25, and the resulting plasmids were then co-transformed into *E*. *coli* BTH101 cells. The diagram shows various plasmid pair combinations (A). Protein-protein interactions were analyzed by the bacterial two-hybrid system, in which the protein interaction strength was indicated by β-galactosidase activity as described (B). Error bars indicate the standard deviation derived from three independently grown cultures.

A number of TCS histidine sensor kinases have been reported to form homodimer [45], and we therefore examined whether RsbK monomer interacts with each other to form a homodimer using BACTH analysis. The reporter plasmids pUT18-*rsbK* and pKNT25-*rsbK* were constructed (S1 Table) to express the RsbK fusion proteins T18-RsbK and NT25-RsbK, comprising the intact signal peptide at the N-terminus, allowing both RsbK fusion proteins to properly integrate into the cytoplasmic membrane of *E*. *coli* BTH101 cells (Fig 4A). As a result, β-galactosidase activity measurements showed that RsbK molecules could strongly interact with each other to form RsbK homodimers (Fig 4B). Additionally, pUT18-*rsbK*_D827N_, pUT18-*rsbK*-*rsbM*-*6xhis* and pUT18-*rsbK*_D827N_-*rsbM*-*6xhis* rather than pUT18-*rsbK* were used to assess the influence of RsbK methylation and the residue replacement at D827 on the formation of RsbK homodimers. Consequently, RsbK methylation and the residue substitution at D827 seemed not to impair RsbK dimerization (Fig 4B).

### Modelling of subdomain interactions in RsbK

**Fig 5:**
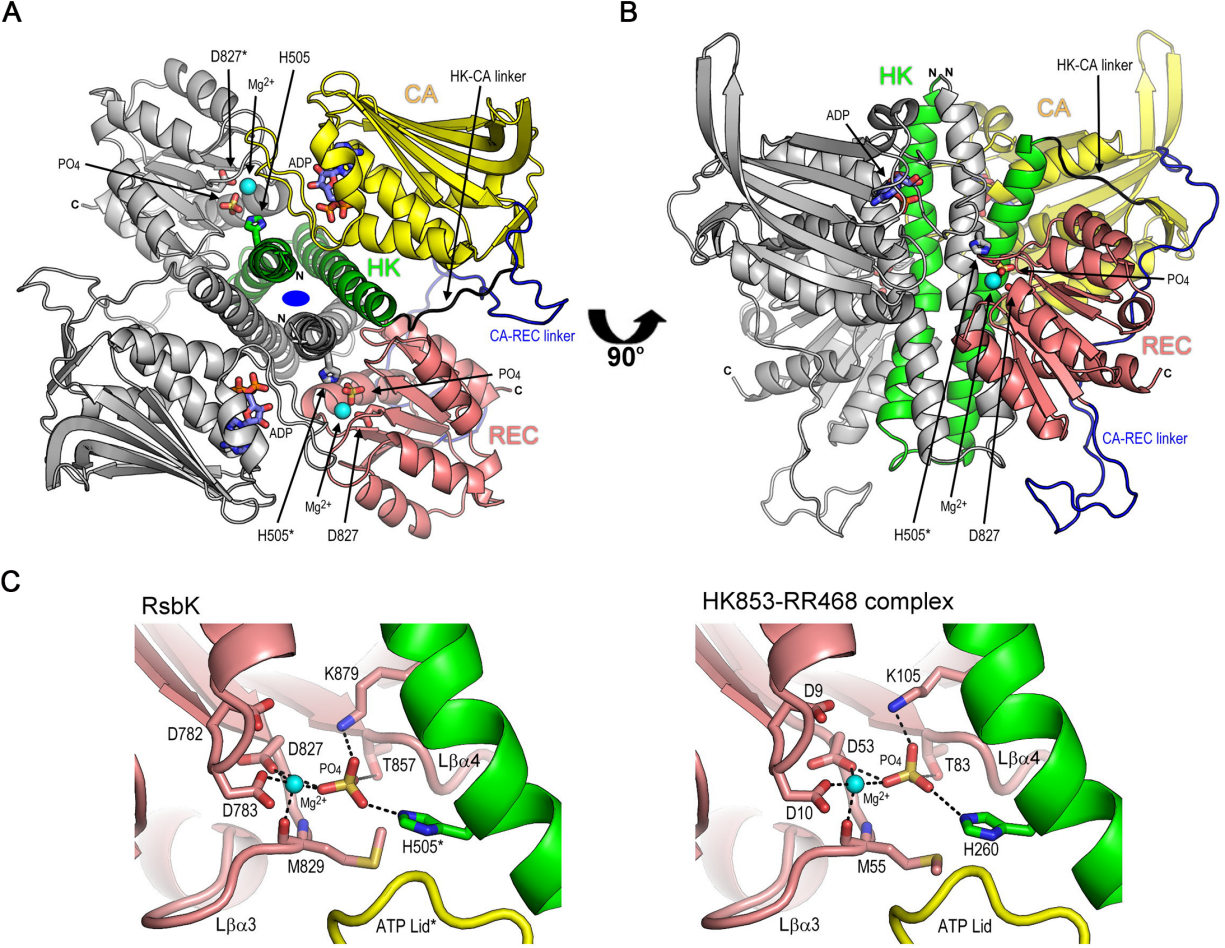
Model structure of the cytosolic portion of the RsbK dimer and detailed views of the interaction between the HK and REC domains. (A and B) Ribbon diagram of the dimer viewed from the cell membrane along the two-fold axis (depicted as a blue oval) (A) or perpendicular to this axis (B), with the cell membrane at the top and the cell interior at the bottom. For clarity, the HK, CA and REC domains in one monomer are colored in green, yellow and pink, respectively. The other monomer is colored in grey, the HK-CA linker is colored in black, and the CA-REC linker is colored in blue. The side chains of the phosphoacceptor H505 and Asp827 residues, as well as the bound phosphate and ADP molecules, are shown as stick models. (C) Comparative view of active sites in the RsbK dimer (left) and in the HK853-RR468 complex (right). The portions belong to the HK, CA and REC domains are colored in green, yellow and pink, respectively. Side chains of active site residues and the phosphate are illustrated as stick models. Mg^2+^ ions are shown as cyan spheres. The ionic bonds involved in Mg^2+^ and phosphate binding are depicted as dashed lines.

To study the function of the REC domain of RsbK from the structural point of view, we took advantage of the resolved crystal structure of the complex formed by the *Thermotoga maritima* class I sensor kinase HK853 and its cognate response regulator, RR468 (PDB ID: 3DGE) [37]. The structure prediction server Phyre2 [38] showed 100% confidence in aligning the sequences of the RsbK HK and REC domains with the template structure, although their amino acid sequences share only 35% identity compared with those of HK853 and RR468. We checked the sequence alignment generated by Phyre2, and confirmed that those functionally important amino acid residues in each RsbK domain are all well aligned (S2 Fig). We concluded that this alignment is valid and started building a complex model using the homology modeling technique MODELLER [36]. The quality of the resulting model (Fig 5) was assessed by PROCHECK [39], and the QMEAN Z-score was calculated [40]. PROCHECK showed that 89% of residues were in favored regions of the Ramachandran plot, and no residues were in disallowed regions. The overall G-factor is -0.05, suggesting that the molecular geometry of the model is acceptable. The Q-MEAN Z-score is a useful measure for identifying significant errors. A Z-score less than -4.0 indicates that any part of the protein structure is not correctly modeled. The Z-score of this model is -0.878, indicating that this model is suitable for subsequent structural analysis.

We built an RsbK HK dimer based on the homodimeric structure of *T*. *maritima* HK853 (Fig 5A and 5B). In the model, the position and orientation of the REC domain to HK dimer was proposed according to those of RR468 with respect to the HK853 dimer in the crystal structure [37]. During model building, we found that the sequence length of the CA-REC linker (residues 732–776) does not allow the program to build a reasonable model of the complex structure in the *cis* binding mode (essentially, an HK domain binds to the REC domain in the same monomer). Therefore, we present the model structure in the *trans* binding mode, in which the HK domain of one monomer interacts with the REC domain from the other monomer.

We used the PISA server [42] to assess the interaction strength between the HK and REC domains in RsbK. The results showed that the interface area is 1239 ± 35 Å^2^. At the interface, there are 13 ± 4 hydrogen bonds and 4 ± 2 salt bridges; the calculated solvation free energy gain upon formation of the interface is approximately -12 ± 2 kcal/mol. PISA calculates a complexation significance score (CSS), which indicates the probability that an interface might represent a 'real' interface on a scale from 0 to 1. The CSS for the HK-REC interface of RsbK is 1.0, suggesting that the interaction between the HK and REC domains may be significant.

## Discussion

One scenario for the function of the REC domain with a putative conserved phospho-acceptor D827 has been proposed to mediate multi-step phosphotransfer reactions because the domain architecture complexity of RsbK resembles those of multi-step phosphorelay TCS sensor kinases [20, 26]. However, three lines of evidence did not support this postulation: (i) typical multi-step phosphotransfer is usually mediated by an Hpt domain or a separate Hpt protein. RsbK does not comprise an intrinsic Hpt domain, and no evidence shows the existence of a distinct Hpt protein based on genomic searching; (ii) the physical interaction between REC-truncated RsbK and RsbY, albeit reduced in strength compared with full-length RsbK [20], retains the possibility of direct phosphotransfer from the conserved H505 of HK domain to the putative phosphor-acceptor D59 in the N-terminal REC domain of RsbY; and (iii) if the REC domain mediates multi-step phosphotransfer reactions, truncation of the REC domain of RsbK or residue substitutions of D827 would halt phosphotransfer and inactivate σ^B^. In fact, truncation of the REC domain of RsbK led to constitutive high σ^B^ activation (Fig 1B), in accordance with the finding of improper σ^B^ induction by REC-truncated RsbK reported by de Been *et al*. [26]. Additionally, the expression of RsbK_D827E_ and RsbK_D827N_ resulting in constitutive high σ^B^ but not expected low σ^B^ expression (Fig 3A). These results reinforce an inhibitory role of the REC domain of RsbK rather than multi-step phosphotransfer, and the residue D827 is functionally required for σ^B^ regulation.

Deletion of the REC domain, the loss of RsbM or expression of a malfunctioning RsbM resulted in constitutive high σ^B^ activation (Fig 1B and 1C), indicating that both REC domain and RsbK methylation were required to maintain the inactive state of the HK domain. BACTH analysis indicated that the S-HK subdomains are the main interfaces for binding to the REC domain (Fig 2-#3, #4, #5, #8, #9), and the interaction strength between the REC domain and REC-CA-HK-truncated RsbK was increased as RsbM was co-expressed (Fig 2-#4, #6). The structural model of RsbK predicts the significant HK-REC interaction (Fig 5). Together, these results suggest that methylation on the S-helix may structurally constrain the S-HK subdomains to interact with the REC domain or to stabilize the REC-HK complex. How S-helix methylation affects REC-HK interaction remains unknown and requires further investigation. In the structural studies of *E*. *coli* Tsr cytoplasmic domain [46–48], the authors found the methylation-mimicking mutant (Q mutant) had lower thermal (B) factors in the adaptation region (the structural equivalent of S-helix). In the crystal structure, each glutamine residue on one helix of the coiled-coil structure of one Tsr Q mutant were at the positions that can make extensive hydrogen bonds to the residues or backbone of the helix in the other Tsr Q mutant in the same dimer. Consequently, the coiled-coil packing was changed and dynamic flexibility reduced. The authors hypothesized that the methylation may stabilize the receptor dimer via forming more hydrogen bonds or possibly favorable hydrophobic interactions involving methyl groups. Based on this proposed mechanism, we conclude our results and conjecture that methylation on the S-helix of RsbK dimer may induces a conformational change on the S-HK subdomains to facilitate the binding of REC domain or to stabilize the REC-HK complex.

A detailed comparison of the active sites in the RsbK dimer and in the HK853-RR468 complex suggests that the HK domain of RsbK may perform a similar phosphotransfer reaction with the REC domain as that found in the HK853-RR468 complex (Fig 5C and S2C Fig). Based on the model proposed by Casino et al. [37, 49], during the phosphotransfer reaction, the conserved Lys (Lys105 in RR468 and Lys879 in RsbK) protruding from the Lβα5 loop of the REC domain binds and neutralizes the negatively charged phosphoryl group being moved. The other key residue in the reaction is the conserved Thr (Thr83 in RR468 and Thr857 in RsbK) at the end of strand β4. In the active site, an Mg^2+^ ion provides a positive charge to assist the phosphotransfer reaction. The conserved Asp residues (Asp9 and Asp10 in RR468 and Asp782 and Asp783 in RsbK) at the Lβα1 loop and the oxygen in the backbone of the conserved Met residue (Met55 in RR468 and Met829 in RsbK) at the Lβα3 loop may coordinate the Mg2^+^ ion during the reaction (S2 Fig). As described above, the structural comparison revealed a similar spatial arrangement of the active site residues in RsbK and HK853-RR468 complex. We therefore propose the active site of RsbK may perform the His-Asp phosphotransfer reaction.

The S-helix in sensor kinases, as an adaptation subdomain, has been reported to function as a switch to prevent the constitutive activation of downstream signaling domains [50]. Moreover, different axial helix rotation states in adaptation subdomain are presumably induced by the upstream HAMP domain and may change the affinity of chemoreceptors to the methylation and demethylation system [51]. Axial helix rotation as a mechanism for signal transduction was first proposed for chemoreceptors [52] and was recently substantiated for adenylyl cyclases [53–55] and histidine kinases [56, 57]. We believe that the interaction between the REC domain and methylated S-HK subdomains is crucial to repress σ^B^. Although RsbK methylation is essential for σ^B^ inhibition, the relief of RsbM-mediated σ^B^ inhibition remains poorly understood. σ^B^ activation is unlikely controlled by demethylation of RsbK because RsbM unpaired to a cognate methylesterase is an orphan methyltransferase, and deletion of the only encoded methylesterase CheB, which is limited to chemotaxis behavior, did not affect σ^B^ regulation in *B*. *cereus* (data not shown). Rather, the expression of D827 mutants caused constitutive high σ^B^ under unstressed conditions (Fig 3A), suggesting that the D827, if phosphorylatable, in the REC domain may play a role in σ^B^ regulation. Because the net charge of D827N (0) is less than that of D827 (-1), our result indicated that loss of the negative charge at D827 appears to increase σ^B^ expression (Fig 3A). In fact, a shift in electric charge at D827 due to alternating phosphorylation and dephosphorylation occurs between phosphorylated D827 (-2) and non-phosphorylated D827 (-1). It seems more likely that the hydrolysis of phosphorylated D827 is required for σ^B^ activation upon environmental stress. We attempted to examine whether H505 or D827 phosphorylation occurs in response to stress. Cell lysates from *B*. *cereus* Δ*rsbKM* complemented with RsbK_Δrec_-Flag and REC-Flag in *trans* with/without heat treatment were subjected to Phos-tag gel electrophoresis followed by Western blotting using an anti-Flag monoclonal antibody. However, the phosphorylation of RsbK_Δrec_-Flag or REC-Flag was hardly detectable in all conditions presumably due to the short half-life of histidyl and aspartidyl phosphate (data not shown). Both RsbK_D827E_ and RsbK_D827N_ resulted in constitutive high σ^B^ inconsistent with the expectation that either D827 variant may activate σ^B^. Actually, different substitutions at a critical residue causing the same biological effect were sometimes observed. For example, reversal frequency was induced by RomR phosphorylation, whereas both RomR_D53E_ and RomR_D53N_ led to lower reversal frequency in *Myxococcus xanthus* [58].

**Fig 6:**
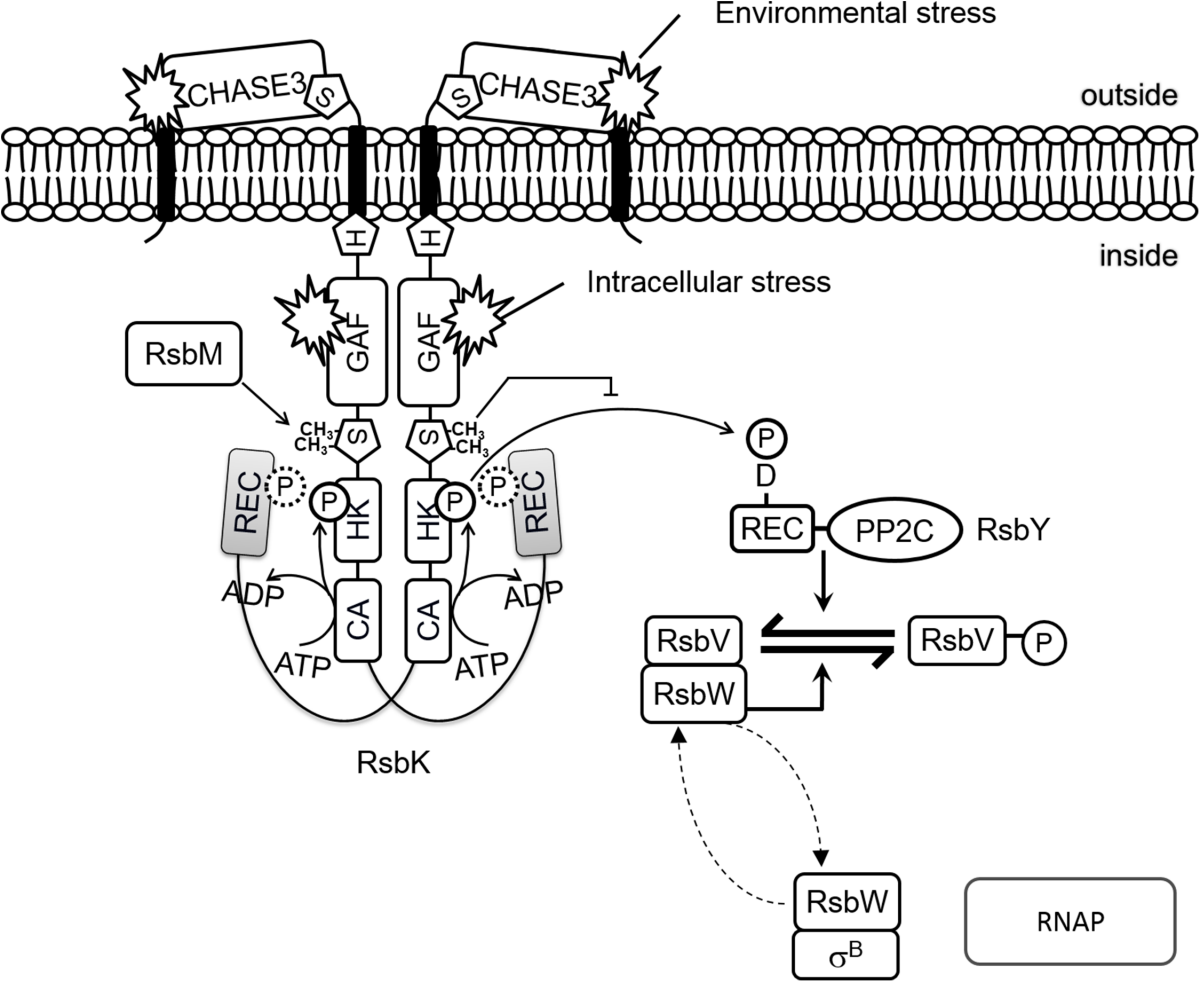
Model for σ^B^ activation in *B*. *cereus*. In the absence of stress, σ^B^ is maintained inactive in a complex with the anti-sigma factor RsbW, which also inactivates RsbV by phosphorylation through its kinase activity. At this stage, RsbM methylates dimeric RsbK on the S-helix required for in *trans* binding of the REC domain to prevent phosphotransfer from H505 to the REC domain of RsbY. If stimulated by specific stress signals, a possible change of the phosphorylation state at D827 (indicated by dashed line), is involved in releases the inhibition of the REC domain, and the phosphorylated H505 of the HK domain becomes accessible for RsbY and increases the phosphorylation of RsbY at N-terminal receiver domain. This action enhances the phosphatase activity of RsbY to hydrolyze RsbV-P. The RsbW:σ^B^ complex is disrupted dephosphorylated RsbV because RsbW forms an alternative complex with RsbV (dashed arrows). Free σ^B^ can now interact with RNA polymerase and direct transcription of the SigB-dependent general stress response genes.

The facts that the REC domain act as a inhibitory element (Fig 2) and RsbK may form a homodimer in *trans* binding mode (Figs 4 and 6) renders RsbK similar to VirA in *Agrobacterium tumefaciens* [26, 59, 60]. Domain organization of VirA consisting of the periplasmic domain perceives sugar and H^+^, the linker domain senses the phenol, the conserved histidine kinase domain, and a C-terminal regulatory receiver domain [61]. Consistent with the finding of the REC domain of RsbK, the receiver domain of VirA has been reported to play a repressive function towards kinase activity of VirA. Deletion of the receiver domain of VirA no longer required phenol for activation and the expression of the receiver domain in *trans* to VirA_△R_ restored the wild-type phenotype [62]. The repressive REC domains of sensor kinases appear not only in VirA and RsbK but also in ArcA in *E*. *coli* [63] and VsrB in *Pseudomonas solanacearum* [64]. It is worth mentioning that although some or possibly all sensor kinases are existed in a homodimeric form in the presence or the absence of inducing stimuli [45], sensor kinases in an oligomeric state are of biological relevance because they are required for signal transduction across membranes and along cytoplasmic domains. Particularly, the membranous VirA intradimer potentially formed an oligomer according to *in vivo* complementation studies [65]; however, whether RsbK can form homodimer or higher degree oligomers and the impact of methylation and D827 mutation on multimerization remains unknown. In addition to some RsbK-type sensor kinases well aligned with RsbK (S2 Fig and S3 Table), 127 RsbK-like sensor kinases were found encoded in 10 bacterial phyla [20]. Thus, the repressive REC domains in sensor kinases interaction with methylated S-helix may be a ubiquitous regulation mechanism in the microbial world.

Given genetic evidence and structure modeling showing the interaction between the S-HK and REC domains of RsbK, we propose the following hypothesis with respect with RsbK-M-Y regulation module (Fig 6): in the upstream signaling cascade, the REC domain of RsbK may act as a regulatory domain of methylated S-HK domains. Upon stimulation, the REC domain may lose its affinity for the HK domain presumably due to the change of state at D827. When released from binding the REC domain, the HK domain becomes active and begins transducing signals via phosphorylation to the downstream RR RsbY at D59 in N-terminal receiver domain.

## Supporting Information

S1 Fig Construction of *B*. *cereus* Δ*rsbKM* and the complementary plasmids. (A) Deletion of the *rsbK-rsbM* operon. A DNA fragment comprising the coding region of a spectinomycin resistance cassette was in-frame inserted into the integration vector pMAD. The constructed plasmid was introduced into *B*. *cereus* via electroporation for allelic exchange. The restriction sites and the inserted DNA length are indicated. (B) Construction of the complementary plasmid pHT304*-rsbK-rsbM* and its derivatives. The DNA fragment, including the partial *bc1009* and *rsbK-rsbM sequences*, was inserted into the vector pHT304 to construct the complementary plasmid pHT304*-rsbK-rsbM*. The other complementary plasmids were constructed by inserting DNA segments lacking either the REC domain or *rsbM*. As described in the experimental procedures, the pHT304-*rsbK*_Δrec_-*recflag* and pHT304-*rsbK*_Δrec_-*recflag*-*rsbM* plasmids were designed to express the Flag-tagged REC domain in *trans*. The restriction sites are indicated.
(TIF)

S2 Fig Sequence features of the HK, CA, and REC domains in RsbK. (A) The HK domain of RsbK compared with that in *T*. *maritima* HK853. (B) CA domain of RsbK compared with that in *T*. *maritima* HK853. (C) REC domain of RsbK compared with that in *T*. *maritima* RR468. The sequences of ten RsbK homologs from different species were also included in the alignments to demonstrate the amino acid conservation of each residue along the RsbK sequence. Information about these RsbK homologs is summarized in S3 Table. Secondary structure elements of HK853 and RR468 are shown below the alignments as helices and arrows for α-helices and β-strands, respectively. In alignment A, the phosphoacceptor H505 is marked by a red triangle. In alignment B, the Mg^2+^-binding Asn625 is marked by a cyan triangle, and residues involved in ATP binding are marked by orange triangles. In alignment C, the phosphoacceptor Asp827 is marked by a magenta triangle, and residues inside the active site are marked by green triangles. Throughout the three alignments, residues participating in van der Waals interactions are marked by orange circles. The residues forming hydrogen bonds and salt bridges are marked by cyan and magenta, respectively.
(TIF)

S1 Table Bacterial strains and plasmids. (DOCX)

S2 Table Oligonucleotides used in this study. (DOCX)

S3 Table Information about the RsbK homologs used in the alignments. (DOCX)
