# Supplementary figures and images for: Methylatable Signaling Helix Coordinated Inhibitory Receiver Domain in Sensor Kinase Modulates Environmental Stress Response in Bacillus Cereus
Source: PLoS One. 2015 Sep 17;10(9):e0137952. doi: 10.1371/journal.pone.0137952 (PMC4574943; doi:10.1371/journal.pone.0137952)

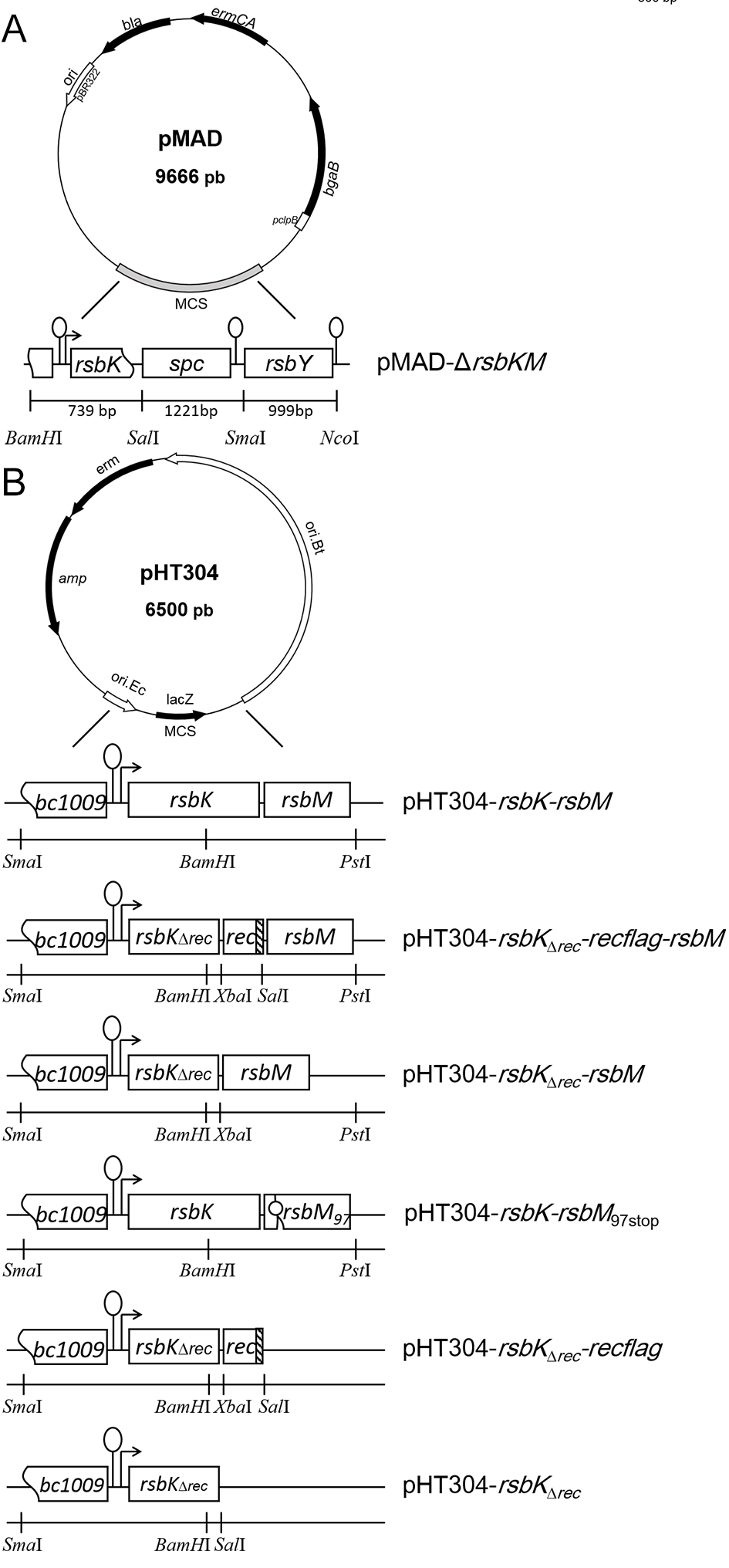

Supplement: S1 Fig — (A) Deletion of the rsbK-rsbM operon. A DNA fragment comprising the coding region of a spectinomycin resistance cassette was in-frame inserted into the integration vector pMAD. The constructed plasmid was introduced into B. cereus via electroporation for allelic exchange. The restriction sites and the inserted DNA length are indicated. (B) Construction of the complementary plasmid pHT304-rsbK-rsbM and its derivatives. The DNA fragment, including the partial bc1009 and rsbK-rsbM sequences, was inserted into the vector pHT304 to construct the complementary plasmid pHT304-rsbK-rsbM. The other complementary plasmids were constructed by inserting DNA segments lacking either the REC domain or rsbM. As described in the experimental procedures, the pHT304-rsbK Δrec-recflag and pHT304-rsbK Δrec-recflag-rsbM plasmids were designed to express the Flag-tagged REC domain in trans. The restriction sites are indicated. (TIF) [file pone.0137952.s001.tif]

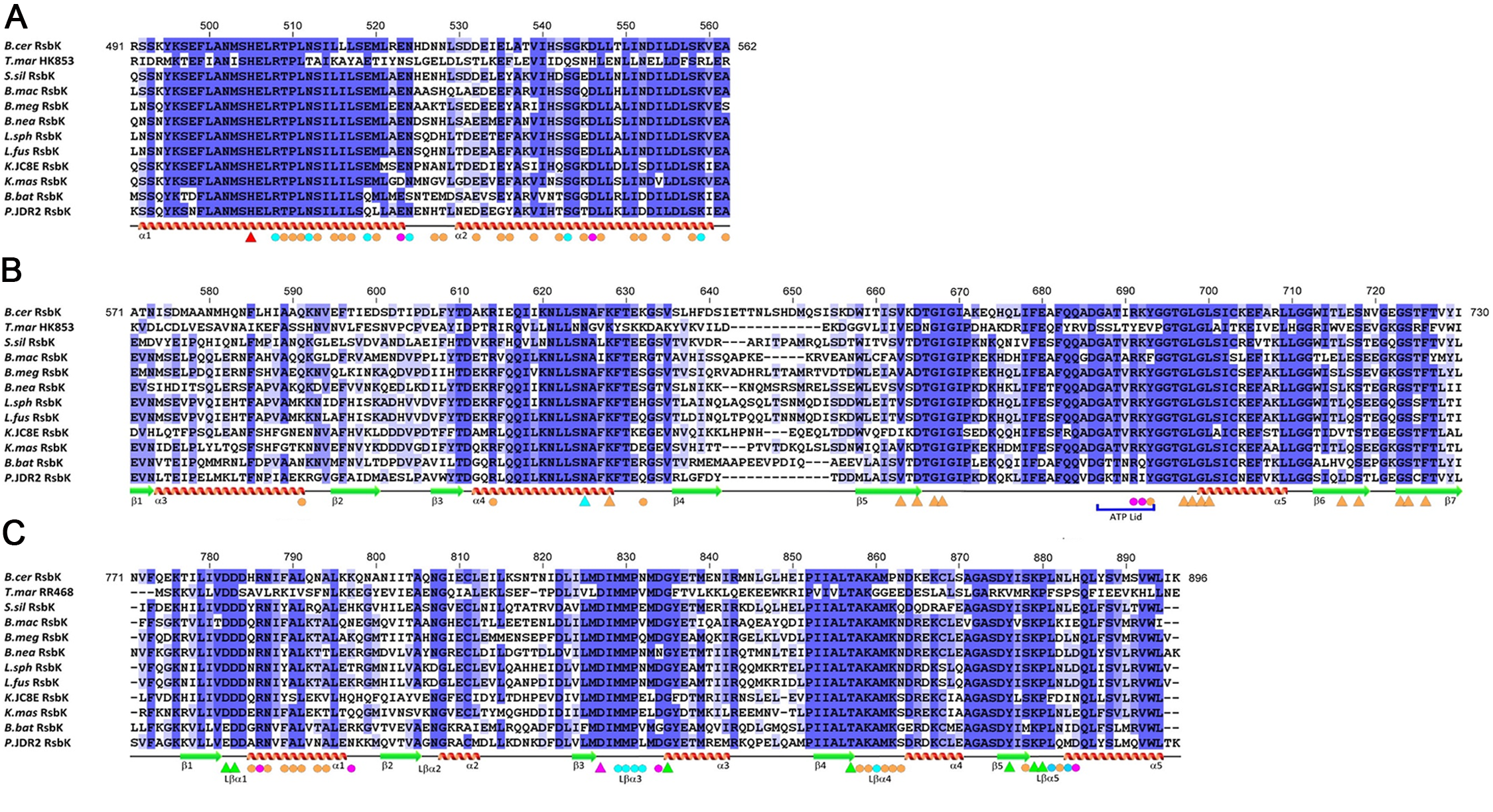

Supplement: S2 Fig — (A) The HK domain of RsbK compared with that in T. maritima HK853. (B) CA domain of RsbK compared with that in T. maritima HK853. (C) REC domain of RsbK compared with that in T. maritima RR468. The sequences of ten RsbK homologs from different species were also included in the alignments to demonstrate the amino acid conservation of each residue along the RsbK sequence. Information about these RsbK homologs is summarized in S3 Table. Secondary structure elements of HK853 and RR468 are shown below the alignments as helices and arrows for α-helices and β-strands, respectively. In alignment A, the phosphoacceptor H505 is marked by a red triangle. In alignment B, the Mg2+-binding Asn625 is marked by a cyan triangle, and residues involved in ATP binding are marked by orange triangles. In alignment C, the phosphoacceptor Asp827 is marked by a magenta triangle, and residues inside the active site are marked by green triangles. Throughout the three alignments, residues participating in van der Waals interactions are marked by orange circles. The residues forming hydrogen bonds and salt bridges are marked by cyan and magenta, respectively. (TIF) [file pone.0137952.s002.tif]
