# Supplementary material for: Methylatable Signaling Helix Coordinated Inhibitory Receiver Domain in Sensor Kinase Modulates Environmental Stress Response in Bacillus Cereus
Source: PLoS One. 2015 Sep 17;10(9):e0137952. doi: 10.1371/journal.pone.0137952 (PMC4574943; doi:10.1371/journal.pone.0137952)
Supplement: S1 Table — (DOCX) [file pone.0137952.s003.docx]

**Supplementary material**

Table S1. Bacterial strains and plasmids

|  |  |  |
| --- | --- | --- |
| **Strain or plasmid** | **Genotype and/or description** | **Source or reference** |
| *E. coli* strains |  |  |
| DH5α | For general purpose cloning | Invitrogen |
| XL-1 blue | For general purpose cloning | Stratagene |
| BL21 (λDE3) | For protein expression | Novagen |
| BTH101 | F-, cya-99, araD139, galE15, galK16, rpsL1 (Str^r^), hsdR2, mcrA1, mcrB1. | Karimova *et al.* ([1998](#_ENREF_3)) |
|  |  |  |
| *B. cereus* strains |  |  |
| ATCC14579 | Wild type | ATCC^a^ |
| Δ*rsbKM* | ATCC14579 *rsbKM::spc^r^* using the vector pMAD-Δ*rsbKM* | This work |
| Plasmids |  |  |
| pMAD | Integration plasmid; *ermC*, *bgaB* | Arnaud *et al.* ([2004](#_ENREF_2)) |
| pMAD-Δ*rsbKM* | Recombinant plasmid for deletion of *rsbKM* using the vector pMAD | This work |
| pDG1728 | Source of *spc^r^* | BGSC^b^ |
| yT&A | TA cloning vector, Ap^r^ | Yeastern Biotech |
| pHT304 | Low copy number vector, Er^r^, Ap^r^ | Arantes & Lereclus ([1991](#_ENREF_1)) |
| pHT304-*rsbK*-*rsbM* | *rsbK-rsbM* gene (3965 bp) and its upstream region (345 bp) was inserted into pHT304, Er^r^, Ap^r^ | This work |
| pHT304-*rsbK*_D827N_*-rsbM* | The *rsbK* in pHT304-*rsbK-rsbM is* replaced by *rsbK*_D827N_ | This work |
| pHT304-*rsbK*_D827E_*-rsbM* | The *rsbK* in pHT304-*rsbK-rsbM is* replaced by *rsbK*_D827E_ |  |
| pHT304-*rsbK*_Δ_*_rec_*-*recflag-rsbM* | *rsbK*_Δ_*_rec_*-*recflag-rsbM* is inserted into pHT304, Er^r^, Ap^r^ | This work |
| pHT304-*rsbK*_Δ_*_rec_-rsbM* | *rsbK*_Δ_*_rec_-rsbM* is inserted into pHT304, Er^r^, Ap^r^ | This work |
| pHT304-*rsbK*-*rsbM*_97stop_ | *rsbK-rsbM_97_* is inserted into pHT304, Er^r^, Ap^r^; *rsbM*_97_ represents replacement of the 97^th^ codon by stop codon | This work |
| pHT304-*rsbK*_Δ_*_rec_-rec-flag* | *rsbK*_Δ_*_rec_-rec-flag* is inserted into pHT304, Er^r^, Ap^r^ | This work |
| pHT304-*rsbK*_Δ_*_rec_* | *rsbK*_Δ_*_rec_* is inserted into pHT304, Er^r^, Ap^r^ | This work |
| pUT18 | Cloning and expression vector; encodes the T18 fragment (CyaA^225–399^); Ap^r^ | Karimova *et al.* ([1998](#_ENREF_3)) |
| pKT25 | Cloning and expression vector; encodes the T25 fragment (CyaA^1–224^); Km^r^ | Karimova *et al.* ([1998](#_ENREF_3)) |
| pKNT25 | Cloning and expression vector; encodes the T25 fragment that is fused in frame downstream from a MCS; Km^r^ | Karimova *et al.* ([1998](#_ENREF_3)) |
| pUT18C-*zip* | A derivative of pUT18C in which the leucine zipper of GCN4 is genetically fused in-frame to the T18 fragment, Ap^r^ | Karimova *et al.* ([1998](#_ENREF_3)) |
| pKT25-*zip* | A derivative of pKT25 in which the leucine zipper of GCN4 is genetically fused in-frame to the T25 fragment, Km^r^ | Karimova *et al.* ([1998](#_ENREF_3)) |
| pUT18-*rsbK* | *rsbK* in pUT18, Ap^r^ | This work |
| pUT18-*rsbK*_D827N_ | *rsbK*_D827N_ in pUT18, Ap^r^ | This work |
| pUT18-*rsbK*^1-770^ | REC truncated *rsbK* in pUT18, Ap^r^ | This work |
| pUT18-*rsbK*^1-609^ | REC-CA truncated *rsbK* in pUT18, Ap^r^ | This work |
| pUT18-*rsbK*^1-495^ | REC-CA-HK truncated *rsbK* in pUT18, Ap^r^ | This work |
| pUT18-*rsbK*^1-411^ | REC-CA-HK-S truncated *rsbK* in pUT18, Ap^r^ | This work |
| pUT18-*rsbK*^1-495^-*rsbM-6xhis* | REC-CA-HK truncated *rsbK* and *rsbM-6xhis* in pUT18, Ap^r^ | This work |
| pUT18-*rsbK*^1-411^-*rsbM-6xhis* | REC-CA-HK-S truncated *rsbK* and *rsbM-6xhis* in pUT18, Ap^r^ | This work |
| pUT18-*rsbK*^412-770^ | CA-HK-S in pUT18, Ap^r^ | This work |
| pUT18-*rsbK*^412-609^ | HK-S in pUT18, Ap^r^ | This work |
| pUT18-*rsbK*^412-495^ | S in pUT18, Ap^r^ | This work |
| pUT18-*rsbK*^412-770^-*rsbM-6xhis* | CA-HK-S and *rsbM-6xhis* in pUT18, Ap^r^ | This work |
| pUT18-*rsbK*^412-609^-*rsbM-6xhis* | HK-S and *rsbM-6xhis* in pUT18, Ap^r^ | This work |
| pUT18-*rsbK*^412-495^-*rsbM-6xhis* | S and *rsbM-6xhis* in pUT18, Ap^r^ | This work |
| pUT18-*rsbK*^496-770^ | CA-HK in pUT18, Ap^r^ | This work |
| pUT18-*rsbK*^496-609^ | HK in pUT18, Ap^r^ | This work |
| pUT18-*rsbK-rsbM-6xhis* | *rsbK* gene and *rsbM-6xhis* in pUT18, Ap^r^ | This work |
| pUT18-*rsbK*_D827N_*-rsbM-6xhis* | *rsbK*_D827N_ gene and *rsbM-6xhis* in pUT18, Ap^r^ | This work |
| pKNT25-*rec* | REC in pKNT25, Km^r^ | This work |
| pKNT25-*rsbK* | *B. cereus rsbK* in pKNT25, Km^r^ | This work |
| pKNT25-*rsbK*_D827N_ | *B. cereus rsbK_D827N_* in pKNT25, Km^r^ | This work |
| pET21b-*rsbK-rsbM-6xHis* | Expression vector, co-expression native from RsbK and His-tagged RsbM, Ap^r^ | This work |
| pET21b-*rsbK* | Expression vector, His-tagged RsbK, Ap^r^ | This work |
| pET21b-*rsbK*_D827N_ | Expression vector, His-tagged RsbK_D827N_, Ap^r^ | This work |
| pET21b-*rsbK*_D827E_ | Expression vector, His-tagged RsbK_D827E_, Ap^r^ | This work |

^a^ATCC：ATCC Biological Resource Center

^b^BGSC：Bacillus Genetic Stock Center

**Reference**

Arantes, O., and Lereclus, D. (1991) Construction of cloning vectors for *Bacillus thuringiensis*. *Gene* **108**: 115-119.

Arnaud, M., Chastanet, A., and Debarbouille, M. (2004) New vector for efficient allelic replacement in naturally nontransformable, low-GC-content, gram-positive bacteria. *Appl Environ Microbiol* **70**: 6887-6891.

Karimova, G., Pidoux, J., Ullmann, A., and Ladant, D. (1998) A bacterial two-hybrid system based on a reconstituted signal transduction pathway. *Proc Natl Acad Sci U S A* **95**: 5752-5756.
