# Supplementary material for: Methylatable Signaling Helix Coordinated Inhibitory Receiver Domain in Sensor Kinase Modulates Environmental Stress Response in Bacillus Cereus
Source: PLoS One. 2015 Sep 17;10(9):e0137952. doi: 10.1371/journal.pone.0137952 (PMC4574943; doi:10.1371/journal.pone.0137952)
Supplement: S2 Table — (DOCX) [file pone.0137952.s004.docx]

**Table S2. Oligonucleotides used in this study**

|  | | |
| --- | --- | --- |
| **Oligonucleotide** | **Sequence (5’–3’)^*^** | **bp** |
| Gene deletion |  |  |
| RsbK-1^st^-*Bam*HI-F | 5’-CTGGATCCTAATGAAACACCTACAATAACG-3’ | 30 |
| RsbK-1^st^-*Sal*I-Rv | 5’-CGGTCGACATCCTCTAATAAAGCAAAAAG-3’ | 29 |
| Spc-Full-*Sal*I-F | 5’-TAAAGTCGACAGTAGTTCACCACCTTTTCC-3’ | 30 |
| Spc-Full-*Sma*I-R | 5’-GTTGCCCGGGTGATTACCAATTAGAATGAA-3’ | 30 |
| RsbKM-3^rd^-*Sma*I-F | 5’-AACCCGGGAAAGCGTATGAAATCCTCTG-3 | 28 |
| RsbKM-3^rd^-*Nco*I-R | 5’-TACCATGGATGCCTGAAATTGATGGACTTG-3 | 30 |
| Bacterial two-hybrid |  |  |
| RsbK-TH-Full-*Pst*I-F | 5’-CGCTGCAGGATGAACTCGAAAGCAAAATT-3’ | 29 |
| RsbK-TH-Full-*Kpn*I-R | 5’-GCGGTACCCGTTTTATTAACCATACACTC-3’ | 29 |
| RsbK-TH-CA-*Kpn*I-R | 5’-TAGGTACCCGATTTGTTTCGGTAGTAACG-3’ | 29 |
| RsbK-TH-HA-*Kpn*I-Rv | 5’-ATGGTACCCGATAAAACAGATCAGGAATCG-3’ | 30 |
| RsbK-TH-S-*Kpn*I-Rv | 5’-TTGGTACCTTGTATTTTGAGCTGCGTAACA-3’ | 30 |
| RsbK-TH-upS-*Kpn*I-R | 5'-TAGGTACCCGATGGATTGTTAAACCTAAATT-3’ | 31 |
| RsbK-TH-S-*Pst*I-F | 5’-AACTGCAGGATGAGTATTATGGGACGTATG-3’ | 30 |
| RsbK-TH-KA-*Pst*I-F | 5’-CACTGCAGGATGAAATCTGAGTTCTTAGCA-3’ | 30 |
| RsbK-18Z-TH-SmaI-R | 5’-TTCCCGGGTTATATCGATTGGCGTTCCAC-3’ | 29 |
| RsbM-6His-TH-SmaI-F | 5’-TTCCCGGGGTGAGGTTTAGTTGGAGTGG-3’ | 28 |
| RsbM-6His-TH-EcoRI-R | 5’-ATGAATTCTCAGTGGTGGTGGTGGTGGTG-3’ | 29 |
| RsbK-REC-*Xba*I-F | 5’-TTTCTAGAGGTTGTTGAAGAAACTATCGTTACT-3’ | 33 |
| Complementary plasmids |  |  |
| Restore-up-*Sma*I-F | 5’-TTCCCGGGTTAATCTCTTTGTAGTATATG-3’ | 29 |
| Restore-up-*Bam*HI-R | 5’-GGATGGATCCTTTTTCTGTGAATTTAAATG-3’ | 30 |
| Restore-do-*Bam*HI-F | 5’-GCAAGGATCCACGGATTGAGCAAATTATTA-3’ | 30 |
| Restore-do-*Pst*I-R | 5’-CGTACTGCAGAACGAATGCGAAATAAAAAG-3’ | 30 |
| RsbK_758stop_-*Xba*I-*Sal*I-R | 5’-TTGTCGACATTTCTAGATTTAGCAGGAATAACATCTTC-3’ | 38 |
| RsbK-16mer-rec-*Xba*I-F | 5’-ATTCTAGATAAGTGAGGTTTAGTTGGAGTGGTTGTTGAAGAAACTATCGTTACTAC-3’ | 56 |
| REC-Flag-*Sal*I-R | 5’-TTGTCGACATTTTTATCATCATCATCTTTATAATCTTTTATTAACCATACACTC-3’ | 51 |
| REC-07atg-*Sal*I-F | 5’-ATGTCGACGTGAGGTTTAGTTGGAGTG-3’ | 27 |
| RsbK_758stop_-07atg-*Xba*I-F | 5’-ATTCTAGATAAGTGAGGTTTAGTTGGAGTG-3’ | 30 |
| Protein over-expression |  |  |
| pET21b-*rsbK*-*NdeI*-F | 5’-CGCATATGAACTCGAAAGCAAAATTTA-3’ | 27 |
| pET21b-*rsbK*-*XhoI*-R | 5’-TCCTCGAGTTTATTTTATTAACCATACAC-3’ | 29 |
| pET21b-*rsbM*-*XhoI*-R | 5’-TGCTCGAGTTTGTATTTTTTGATAAATTT-3’ | 29 |
|  |  |  |
| Site-directed mutagenesis |  |  |
| *rsbK*_D827N_-F | 5’-CCTTATTTTAATGAATATTATGATG-3’ | 25 |
| *rsbK*_D827N_-R | 5’-TTCGGCATCATAATATTCATTAAAA-3’ | 25 |
| *rsbK*_D827E_-F | 5’-ACAAATATTGACCTTATTTTAATGGAA-3’ | 27 |
| *rsbK*_D827E_-R | 5’-CGGCATCATAATTTCCATTAAAATAAG-3’ | 27 |
| *rsbM*_97end_-F | 5’-TGCCTTTTTTTAAGCGCTAA-3’ | 20 |
| *rsbM*_97end_-R | 5’-TCTCTTAGCGCTTAAAAAAA-3’ | 20 |

*Introduced restriction sites are underlined.
