# Supplementary material for: Methylatable Signaling Helix Coordinated Inhibitory Receiver Domain in Sensor Kinase Modulates Environmental Stress Response in Bacillus Cereus
Source: PLoS One. 2015 Sep 17;10(9):e0137952. doi: 10.1371/journal.pone.0137952 (PMC4574943; doi:10.1371/journal.pone.0137952)
Supplement: S3 Table — (DOCX) [file pone.0137952.s005.docx]

**Table S3.** Information about the RsbK homologs used in the alignments.

| **Protein** | **Organism** | **NCBI accession number** |
| --- | --- | --- |
| *B. cer* RsbK | *Bacillus cereus* | WP_002197482.1 |
| *S. sil* RsbK | *Solibacillus silvestris* StLB046 | YP_006461545.1 |
| *B. mac* RsbK | *Bacillus macauensis* | WP_007201197.1 |
| *B. meg* RsbK | *Bacillus megaterium* WSH-002 | YP_005495874.1 |
| *B. nea* RsbK | *Bacillus nealsonii* | WP_016201617.1 |
| *L. sph* RsbK | *Lysinibacillus sphaericus* C3-41 | YP_001700276.1 |
| *L. fus* RsbK | *Lysinibacillus fusiformis* | WP_004233472.1 |
| *K.* JC8E RsbK | *Kurthia* sp. JC8E | WP_010304432.1 |
| *K. mas* RsbK | *Kurthia massiliensis* | WP_010289398.1 |
| *B. bat* RsbK | *Bacillus bataviensi* | WP_007086098.1 |
| *P.* JDR2 RsbK | *Paenibacillus* sp. JDR-2 | YP_003010574.1 |
